# Supplementary material for: MetaRibo-Seq measures translation in microbiomes
Source: Nat Commun. 2020 Jun 29;11:3268. doi: 10.1038/s41467-020-17081-z (PMC7324362; doi:10.1038/s41467-020-17081-z)
Supplement: Supplementary file 10 — Supplementary Data 7 [file 41467_2020_17081_MOESM10_ESM.zip › File2/Confidence_High_Taxonomy/416479_out.krona.html]

Javascript must be enabled to view this page.

members
magnitude
magnitudeUnassigned
count
unassigned
taxon
rank

416479\_out

4

2
superkingdom
4

phylum
4
1239

class
4
186801

4
186802

SRS148159\_contig\_number\_32035
1
order

3
family
541000

3
genus
1263


SRS015217\_contig\_number\_32019SRS017521\_contig\_number\_32445SRS146813\_contig\_number\_26341
1816688
species
3
